# Cardiac conduction system abnormalities in ankylosing spondylitis: a cross-sectional study

**DOI:** 10.1186/1471-2474-14-237

**Published:** 2013-08-12

**Authors:** Helena Forsblad-d’Elia, Hanna Wallberg, Eva Klingberg, Hans Carlsten, Lennart Bergfeldt

**Affiliations:** 1 Institute of Medicine, Department of Rheumatology and Inflammation Research, Sahlgrenska Academy, University of Gothenburg, Box 480, S-405 30, Gothenburg, Sweden; 2 Institute of Medicine, Molecular and Clinical Medicine/Cardiology, Sahlgrenska Academy, University of Gothenburg, Gothenburg, Sweden

**Keywords:** Ankylosing spondylitis, Spondyloarthritis, Heart disease, Inflammation, Disease activity, HLA B27

## Abstract

**Background:**

Cardiac conduction disturbances are common in spondyloarthropathies such as ankylosing spondylitis (AS). Whether their occurrence can be linked to signs and symptoms of rheumatic disease activity is an unsettled issue addressed in this study.

**Methods:**

In this cross-sectional study patients with AS according to modified New York criteria but without psoriasis, inflammatory bowel disease, dementia, pregnancy, other severe diseases such as malignancy and difficulties in answering questionnaires were invited; and 210 participated (120 men), mean age 49 years (SD 13; range: 16–77). Questionnaires, physical examination, ECG, and laboratory tests were performed at the same visit.

**Results:**

Cardiac conduction disturbances were common and diagnosed in 10-33%, depending on if conservative or less conservative predefined criteria were applied. They consisted mostly of 1^st^ degree atrio-ventricular block and prolonged QRS duration, but one patient had a pacemaker and 7 more had complete bundle branch blocks. Conduction abnormalities were associated mainly with age, male gender and body weight, and not with laboratory measures of inflammation or with Bath Ankylosing Spondylitis Disease Activity Index. Neither were they associated with the presence of HLA B27, which was found in 87% of all patients; the subtype B270502 dominated in all patients.

**Conclusions:**

Cardiac conduction abnormalities are common in AS, but not associated with markers of disease activity or specific B27 subtypes. Even relatively mild conduction system abnormalities might, however, indirectly affect morbidity and mortality.

## Background

Chronic inflammatory disease has recently come into focus as a risk factor for the development of cardiovascular dysfunction including coronary artery disease (CAD) [1,2]. Increased cardiovascular morbidity and mortality seems indisputable in rheumatoid arthritis and systemic lupus erythematosus seems to predispose for premature CAD, especially in younger women [2]. The situation is less clear for ankylosing spondylitis (AS) and the other spondylarthropathies (SpA) although cardiovascular diseases are the leading cause of death just as in the general population [2-4], and there is data demonstrating an increased risk of cardiovascular disease also in AS patients [5-7]. However, in a Brazilian registry study on SpA cardiac manifestations were infrequently found [8].

There is a strong immuno-genetic link between spondylarthropathies, especially AS, and the Human Leukocyte Antigen (HLA) B27 and the associated inflammatory process might not only cause rheumatic disease but may also target cardiac function [9]. Typically cardiac involvement consists of conduction system abnormalities and/or aortic valve insufficiency which might require pacemaker therapy and valve replacement, respectively. Obliterative (occlusive) endarteritis of small vessels supplying the aortic root and the atrio-ventricular (AV) node is a salient histological feature just as was described in the vicinity of afflicted joints more than 50 years ago [9,10]. The inflammatory process includes development of fibrosis, which according to one autopsy study might contribute to cardiac involvement [11].

In the present study we focus on patients with AS fulfilling the modified New York criteria’s for AS [12] since AS is a more homogeneous disease contrary to SpA comprising different phenotypes. Furthermore, and in contrast to some previous observations from SpA patients in general [9], a recent study focusing specifically on AS patients reported a relation between rheumatic disease characteristics and both AV and intra-ventricular conduction intervals [13]. Although other factors such as age and gender also were associated with signs of cardiac involvement the data might be interpreted as supportive of a progressive inflammatory process including fibrosis. Such data – if confirmed - have potential mechanistic and therapeutic implications. The present study was therefore initiated to test whether the Dutch results were reproducible in a cross-sectional study of a Swedish cohort of AS patients. Furthermore, we assessed HLA B27 subtypes to explore any relation between cardiac involvement and specific subtypes.

## Methods

**Figure 1:**
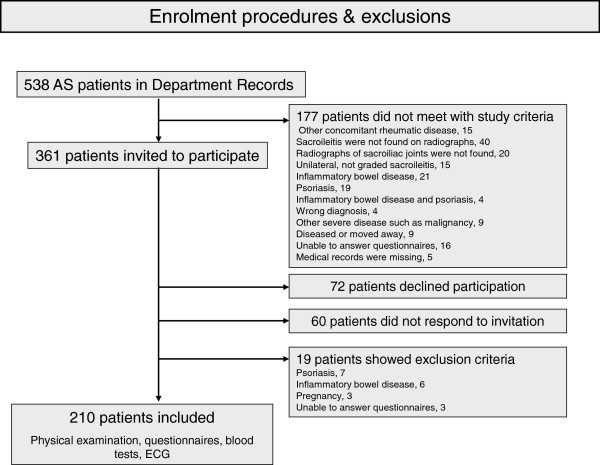
Flow-chart describing the enrollment of the study cohort.

The study cohort consists of 210 patients (120 men) with mean age (SD; range) 49 (13; 16–77) years. They were identified through the patient records at three different departments of Rheumatology in Sweden, in Gothenburg (Sahlgrenska University Hospital), Borås and Alingsås. All patients fulfilled the modified New York criteria’s for AS [12]. Exclusion criteria were psoriasis, inflammatory bowel disease, dementia, pregnancy, other severe diseases such as malignancy and difficulties in answering questionnaires. The medical records of all AS patients registered in the hospitals’ databases were reviewed. The flow-chart for enrolment is shown in Figure 1. All patients meeting study criteria were asked to participate in this cross-sectional AS study investigating in particular some cardiac and skeletal consequences of the disease. For further information about the study design see also a previous report [14]. This report focuses on results of electrocardiographic (ECG) recordings and the association between cardiac conduction system abnormalities and markers of disease activity and HLA B27 subtypes. The study was performed according to the Helsinki Declaration of 1975, revised in 1983, approved by the Regional Ethics Committee in Gothenburg and the patients gave written informed consent to participate. The 151 patients who declined to participate or were excluded were on average slightly younger (46 ± 13 years), but the sex distribution was the same among included and not included patients.

### Electrocardiographic recording and analysis

Standard resting 12-lead ECG were recorded at a paper speed of 50 mm/s, on the same occasion as blood samples were drawn, physical examination performed and questionnaires completed. These ECG’s were then read first by one author (HW) and then jointly by two authors (HW, LB), who were unaware of patient characteristics. Standard criteria used in clinical routine were used primarily: 1^st^ degree AV-block was diagnosed in the presence of a PQ-interval (= PR interval) ≥ 220 ms (0.22 s) and a broad QRS complex when the duration was ≥ 120 ms (0.12 s). In addition, and in order to allow comparison with the Dutch study, less conservative criteria used by them were applied also in this study [13], i.e. 1^st^ degree AV-block was tentatively diagnosed in the presence of a PQ interval of ≥ 200 ms (0.20 s) and a “prolonged” QRS interval ≥ 100 ms (0.10 s). Like in the Dutch study the PR and QRS intervals were analysed as continuous variables and dichotomized at specified threshold values.

### Rheumatologic evaluation and blood analyses

The patients underwent physical examination by the same physician (EK), including Bath Ankylosing Spondylitis Metrology Index (BASMI), and answered questionnaires about medical history, medication, Bath Ankylosing Spondylitis Disease Activity Index (BASDAI), and Bath Ankylosing Spondylitis Functional Index (BASFI) [15].

The presence of the HLA-B27 antigen and its alleles HLA-B2701 to HLA-B2738 were assessed by HLA typing with sequence-specific oligonucleotide primers (PCR-SSO) by LABType®, (One Lambda, Inc, CA, USA) and use of the Luminex platform at the Department of Clinical Immunology and Transfusion Medicine, Sahlgrenska University Hospital.

Erythrocyte sedimentation rate, C-reactive protein, haemoglobin, white blood cell count, and platelet count were analysed consecutively by standard laboratory techniques at the Departments of Clinical Chemistry.

### Statistical methods

Mean and standard deviation (SD) was used for descriptive purposes. Univariate and forward stepwise multivariate linear and logistic regression analysis was used to assess associations between the occurrence of conduction abnormalities and ECG measures with clinical, anthropometric and laboratory data. Chi-square test or Fisher’s exact two-tailed test were used for comparison of proportions. A p-value < 0.05 was considered significant. SAS (version 9.1, SAS Institute Inc., Cary, NC, USA) and STATISTICA (version 10, StatSoft, Inc, Tulsa, OK, USA) were used in the analyses.

## Results

Demographic data and clinical characteristic of the 210 AS patients included in the study is presented in Table 1. A majority (77%) of the patients were treated with non-steroidal anti-inflammatory drugs, while specific anti-rheumatic agents and analgesics were less common and received by 37 and 26%, respectively. Beta-blockers were the most common cardiovascular drug; see Table 1 for details.

**Table 1:** Clinical characteristics for patients with ankylosing spondylitis participating in the study (n = 210)


Age (years)	49 (13)
Men/Women	120/90
BMI	26.0 (4.5)
Smoker ≥ 6 months	105 (50%)
Rheumatologic characteristics	
Age at symptom onset (years)	26 (10)
Symptom duration (years)	24 (13)
Diagnostic age (years)	36 (11)
Diagnostic duration (years)	14 (11)
BASDAI (score)	3.6 (2.1)
BASFI (score)	2.7 (2.1)
BASMI (score)	3.1 (1.6)
Blood tests	
HLA B27 n (%)	183* (87)
Hemoglobin (g/L)	139 (13)
White blood cell count (×10^9^/L)	7.0 (2.2)
Platelet count (×10^9^/L)	298 (75)
Erythrocyte sedimentation rate (mm/h)	15 (14)
C-reactive protein (mg/L)	8.4 (10.2)
Concomitant diseases	
Diabetes mellitus	6 (2.9)
Hyperlipidemia	17 (8.1)
Hypertension	56 (27)
Other cardiovascular diseases¶	26 (12)
Coronary artery disease	6 (2.9)
Heart failure	4 (1.9)
Valvular disease	6 (2.9)
Arrhythmia	4 (1.9)
Various not specified/	10 (4.8)
Stroke	4 (1.9)
Medication	
ACEI/ARB	23 (11)
Analgesics	54 (26)
Antiarrhythmic agents	3 (1.4)
Antidiabetic agents	3 (1.4)
Antihypertensive agents	15 (7.1)
ASA	22 (11)
Betablockers	28 (13)
Calcium antagonists	7 (3.3)
Digitalis	2 (1.0)
Diuretics	15 (7.1)
DMARD	77 (37)
Lipid modulators	15 (7.1)
NSAID	161 (77)
TNF inhibitor	45 (21)
Warfarin	2 (1.0)

*11 were homozygous.

¶more than one is possible.

Data regarding demographic, clinical and laboratory analyses are presented as mean (SD), while concomitant diseases and medication are presented in numbers (%).

*Abbreviations*: *ACEI* angiotensin converting enzyme inhibitor, *ARB* angiotensin II receptor blocker, *ASA* acetylsalicylic acid, *BMI* body mass index, weight/height^2^, *BASDAI* Bath Ankylosing Spondylitis Disease Activity Index, *BASFI* Bath Ankylosing Spondylitis Functional Index, *BASMI* Bath Ankylosing Spondylitis Metrology Index, *DMARD* disease modifying antirheumatic drug, *NSAID* non-steroidal anti-inflammatory drugs, *TNF* tumour necrosis factor.

### Heart rhythm

The mean (SD) resting heart rate was 66 (12) beats/min; 205 had sinus rhythm, two had atrial fibrillation, one patient’s rhythm alternated between sinus and junctional (AV nodal) rhythm, one had ectopic atrial activity, and one pacemaker rhythm. There were 10 patients with bradycardia (< 50 beats/min) and 4 with tachycardia (> 100 beats/min); the minimum heart rate was 44 and the maximum 112 beats/min.

### Blood pressure

The systolic blood pressure was on average 135 mm Hg (SD 20; range 95–190) and the diastolic pressure 77 mm Hg (SD 10; range 50–110); 69 had a systolic pressure > 140 mm Hg, which in 17 of them was combined with a diastolic pressure ≥ 90 mm Hg (8 of them > 90), while two had a diastolic pressure of 90 mm Hg and normal systolic pressures.

### Atrio-ventricular and intra-ventricular conduction

The PQ interval was on average 164 (27) ms. First degree AV-block defined as a PQ interval ≥220 ms was present in 7 patients (3.3%); and in 19 (9%) when defined as a PQ interval ≥200 ms (as in ref. [13]). One patient (0.5%) had a pacemaker, but otherwise no high (2^nd^ or 3^rd^) degree AV-blocks were observed. A broad QRS complex (≥120 ms) was observed on ECG’s from 7 patients, two had typical right bundle branch block (RBBB; one in combination with a left anterior fascicular block, LAFB), one had typical and another atypical left bundle branch block (LBBB), one had a pacemaker and two had severe intra-ventricular conduction abnormalities without typical bundle branch block pattern. When using a QRS duration ≥100 ms as a criterion of “prolonged” QRS (as in ref. [13]), 57 patients (27%) fell into this category, Table 2. Nine patients had isolated block in the left anterior fascicle (LAFB; apart from the one above with RBBB). Altogether 21 patients had atrio-ventricular and/or intra-ventricular conduction abnormalities according to conservative criteria (10%; 95% CI: 5.9-14.1); see Table 3 for details. When adding first those 10 with a PQ interval of 200–219 ms the number increased to 31 (14.7%; 95% CI: 11.0-18.4) and then those with a QRS interval of 100–119 ms there were 39 more patients, altogether 70 patients (33.3%; 95% CI: 26.9-39.7). While some ECG specialists apply age differentiated PR intervals for a diagnosis of 1^st^ degree AV-block even in an adult cohort, there is consensus about the criterion for broad QRS, which is ≥120 ms (0.12 s). This is the reason why we dichotomized the PQ interval at two threshold values and the QRS interval at only one in the analyses below.

**Table 2:** Results of electrocardiographic analysis in patients with ankylosing spondylitis (n = 210)


Heart rate (beats/min)	66 (12)
RR-interval	930 (159)
P-wave duration**	97 (15)
PQ-interval*	164 (27)
QRS-interval**	88 (14)
QRS-interval <100*	153 (73%)
QRS-interval 100-119*	50 (24%)
QRS-interval ≥120*	7 (3.3%)
QTcB-interval**	406 (29)
Atrial fibrillation	2 (1.0%)
AV-block, 1^st^ degree (≥220)	7 (3.3%)
AV-block, 1^st^ degree (≥200)	19 (9%)
AV-block, 2^nd^ degree	0
AV-block, 3^rd^ degree	0
Left anterior fascicular block	12 (6%)
Pacemaker	1 (0.5%)
Any (≥1) conduction abnormality	21 (10%)
with AV-block, 1^st^ degree (≥200)	31 (15%)

* n = 209 (pacemaker patient excluded).

** n = 207 (pacemaker and atrial fibrillation patients excluded).

Data are presented as mean (SD) or as otherwise stated. All intervals are in ms.

**Table 3:** Characteristics of ankylosing spondylitis patients with cardiac conduction abnormalities according to conservative criteria; PQ-interval ≥ 220 ms and/or QRS complex ≥ 120 ms (n = 21)

**Age**	**Sex**	**Symptom duration**	**IVCD**	**AV block**	**BAS DAI**	**BASFI**	**BAS MI**	**HLA B27 alleles**	**Cardio-active Rx**	**CVD**
				**PM**						
30	m	8	LAFB		5.76	3.2	3	1	0	
38	m	18		AV I	2.99	1.34	1.6	1	0	
43	m	14	LBBB		1.95	1.07	1.4	1	0	
50	m	27		AV I	5.03	3.45	2.8	1	0	
52	m	13		AV I	3.9	2.43	2	1	0	HT
57	m	33	RBBB	AV I	3.4	2.3	3.4	1	0	Valve disease
			LAFB							
59	m	36	LAFB		1	0.9	5.6	1	0	
61	m	34		AV I	1	1	1.8	1	BB*	HT
62	m	38	LAFB		4.65	3.88	3.6	1	(BB)	Valve disease,
										CAD
63	m	36	LAFB		1.38	3.69	7.2	1	0	HT
63	m	49		AV I	4.11	4.26	6	1	0	
64	m	45	LAFB		0.89	2.36	6.8	1	0	Valve disease
64	m	44	LAFB		2.34	2.53	7	1	(BB)	AF; HT
68	m	39	LAFB		3.44	4.99	4.2	0	0	Valve disease
68	m	42	RBBB		5	4.56	5.6	1	0	Valve disease
										HT
71	m	55	LAFB		7.1	8.7	3.4	1	(BB)	HT
74	m	25	LBBB		NA	NA	6	1	(BB)	Valve disease,
										heart failure
75	m	52		PM	3.84	6.65	7.2	1	(BB)	Valve disease
										HT
77	m	23	LAFB		6.78	7.9	6.8	1	(BB)	Valve disease
										HT
46	f	39		AV I	2.73	1.62	2.8	1	0	
62	f	46	LAFB		3.57	3.09	3.2	2	0	

Noted in the right hand column are substances that might affect atrio- and/or intra-ventricular conduction; when within brackets no clinical effect is assumed in the patient.

*Abbreviations*: *AI* aortic valve insufficiency, *AV* atrio-ventricular, *AV I* 1^st^ degree AV block, BASDAI, BASFI, and BASMI1, see Table 2, *BB* beta-blocker, *CAD* coronary artery disease, *CVD* cardio-vascular disease, *HT* hypertension, *IVCD* intra-ventricular conduction disturbances, *LAFB* left anterior fascicular block, *LBBB* left bundle branch block, *NA* not available//missing data, *PM* pacemaker, *RBBB* right bundle branch block.

* beta-blocker therapy might impair atrio-ventricular but not intra-ventricular conduction.

### Comparison between clinical and ECG data

We first compared the group of 21 patients with conduction abnormalities according to conservative criteria (A) with the remaining 189, and then the group of 31 patients including also the 10 with 1^st^ degree AV-block diagnosed based on a PQ interval of 200–219 ms (B) with the remaining 179 patients. Multivariate analysis showed that age (p < 0.001) and male sex (p < 0.01) were independent determinants for group A, while symptom duration (p < 0.001) and body weight (p < 0.0001) were determinants for group B.

Furthermore, multivariate linear regression analysis showed a relation between the duration of the PQ interval and age (p < 0.001, male sex (p < 0.01), and body weight (p = 0.02), and an inverse relation to leukocyte count (p < 0.01). The QRS duration was similarly related to male sex (p = 0.001) and body weight (p < 0.01).

The numerical data of these analyses appear in Tables 4 and 5.

**Table 4:** Univariate associations between cardiac involvement and clinical data in 210 patients with ankylosing spondylitis

	**PQ interval**		**QRS interval**		**AVB IVCD**		**AVB IVCDx**	
	**Beta (95% CI)**	**p**	**Beta (95% CI)**	**p**	**OR (95% CI)**	**p**	**OR (95% CI)**	**p**
Age	0.47 ( 0.19,0.74)	.001	0.10 (−0.04,0.25)	.17	1.088 (1.040,1.138)	.0003	1.070 (1.033,1.109)	.0002
Male gender	15.3 (8.2,22.4)	<.0001	10.8 (7.3,14.3)	<.0001	8.28 (1.88,36.53)	.005	4.70 (1.73,12.80)	.002
Age spt onset	0.27 (−0.12,0.66)	.17	0.12 (−0.08,0.32)	.24	1.002 (0.956,1.051)	.92	1.015 (0.976,1.055)	.46
Symptom dur.	0.38 ( 0.09,0.67)	.01	0.02 (−0.13,0.17)	.79	1.069 (1.030,1.111)	.0005	1.055 (1.023,1.088)	.0007
BASDAI	−0.16 (−0.88,1.56)	.86	−0.93 (−1.78,-0.09)	.03	0.993 (0.801,1.232)	.95	0.997 (0.832,1.194)	.97
BASFI	1.68 (−0.09,3.46)	.06	−0.44 (−1.33,0.45)	.33	1.207 (0.984,1.479)	.07	1.239 (1.041,1.474)	.02
BASMI	3.42 ( 1.12,5.72)	.004	0.24 (−0.95,1.44)	.69	1.593 (1.224,2.074)	.0005	1.566 (1.241,1.975)	.0002
Length	−0.61 ( 0.24,0.97)	.001	0.52 ( 0.35,0.70)	<.0001	1.060 (1.009,1.114)	.02	1.048 (1.006,1.092)	.02
Weight	0.52 ( 0.31,0.74)	<.0001	0.29 ( 0.18,0.40)	<.0001	1.039 (1.012,1.067)	.005	1.051 (1.025,1.077)	<.0001
BMI	0.66 (−0.14,1.46)	.11	0.40 (−0.01,0.81)	.05	0.956 (0.857,1.068)	.43	1.011 (0.931,1.097)	.80
Hb	0.57 ( 0.29,0.85)	<.0001	0.27 ( 0.13,0.42)	.0002	1.027 (0.989,1.066)	.17	1.038 (1.005,1.073)	.02
ESR	−0.09 (−0.36,0.18)	.49	−0.10 (−0.24,0.03)	.14	1.007 (0.977,1.038)	.65	0.991 (0.959,1.023)	.56
CRP	−0.04 (−0.41,0.33)	.82	−0.04 (−0.22,0.15)	.69	1.025 (0.994,1.058)	.12	1.021 (0.992,1.052)	.16
WBC	−1.79 (−3.51,-0.07)	.04	−0.56 (−1.43,0.32)	.21	0.977 (0.787,1.213)	.83	0.863 (0.700,1.063)	.17
PLT	−0.05 (−0.10,-0.00)	.05	−0.01 (−0.04,0.01)	.37	0.998 (0.992,1.004)	.58	0.995 (0.990,1.001)	.10
B27 alleles	6.2 (−4.8,17.2)	.27	−0.2 (−5.9,5.4)	.93	3.21 (0.41,24.95)	.26	2.37 (0.53,10.55)	.26

AVB IVCD denotes atrio-ventricular and intra-ventricular conduction disturbances according to standard (conservative) criteria (n = 21).

AVB IVCDx denotes such disturbances according to less conservative criteria (n==31) for comparison with ref. [13].

*Abbreviations*: PQ interval = duration from P-wave to QRS complex; QRS interval = duration of QRS complex; AVB atrio-ventricular Block; IVCD intra-ventricular conduction disturbances: BASDAI Bath Ankylosing Spondylitis Disease Activity Index; BASFI Bath Ankylosing Spondylitis Functional Index; BASMI Bath Ankylosing Spondylitis Metrology Index; BMI Body Mass Index; Hb Hemoglobin (g/L); ESR Erythrocyte Sedimentation Rate (mm/h); CRP C-reactive protein (mg/L); WBC White Blood Cells (×10^9^/L); PLT Platelet Count (×10^9^/L).

Beta regression coefficient; CI confidence interval; OR = odds ratio.

Univariate associations (univariate linear regression and logistic regression).

**Table 5:** Multivariate analyses between cardiac involvement and clinical data in 210 patients with ankylosing spondylitis

	**PQ interval**		**QRS interval**		**AVB IVCD**		**AVB IVCDx**	
	**Beta (95% CI)**	**p**	**Beta (95% CI)**	**p**	**OR (95% CI)**	**p**	**OR (95% CI)**	**p**
Age	0.48 (0.21,0.74)	.0005			1.091 (1.042,1.143)	.0002		
Symptom dur.							1.062 (1.026,1.098)	.0003
Weight	0.32 (0.06,0.57)	.02	0.18 (0.05,0.31)	.007			1.055 (1.027,1.083)	<.0001
WBC	−2.12 (−3.70,-0.53)	.009						
Male gender	10.2 (2.1,18.3)	.01	7.0 (2.8,11.2)	.001	9.64 (2.12,43.87)	.003		

AVB IVCD denotes atrio-ventricular and intra-ventricular conduction disturbances according to standard (conservative) criteria (n = 21).

AVB IVCDx denotes such disturbances according to less conservative criteria (n = 31) for comparison with ref. [13].

PQ interval = duration from P-wave to QRS complex; QRS interval = duration of QRS complex; AVB = atrio-ventricular Block; IVCD intra-ventricular conduction disturbances; WBC White Blood Cells (×10^9^/L).

Beta regression coefficient; CI confidence interval; OR = odds ratio.

Multivariate analyses (forward stepwise multivariate linear regression and logistic regression).

HLA B27 was present in 183 patients (87%); 176 of them had the subtype B270502 (8 were homozygous for this subtype and 2 had other B27 subtypes as well). B2702 was the only B27 subtype in 6 patients and B270401 in the remaining patient. At least one B27 allele was found in 20 of 21 (95%) patients with vs. 163 of 189 (86%) without cardiac conduction abnormalities (NS). Applying the less conservative criteria the corresponding figures were 26 out of 31 vs. 157 out of 179 (NS).

Group A patients (n = 21) received significantly more cardiovascular pharmacotherapy than the remaining 189 patients, although there might be several indications for a specific substance group; examples are beta-blockers (33 vs. 11%; p = 0.01), ACE-inhibitors/angiotensin II receptor antagonists (38 vs. 8%; p < 0.001), diuretics and lipid modulators (both 24% vs. 5%; p < 0.01) and ASA (33 vs. 8%; p < 0.01). There were no significant differences for calcium antagonists and other agents with antihypertensive action apart from those mentioned above.

Pharmacotherapy that might contribute to conduction disturbances are specified for each patient in Table 3. In only one case such therapy might have contributed to a 1^st^ degree AV-block (the 61 years old man in Table 3).

## Discussion

In this Swedish cohort of 210 AS patients ECG abnormalities were common and cardiac conduction system abnormalities were found in 10% according to conservative criteria and 33% when less conservative criteria were applied. Their presence was, however, associated mainly with age, male gender and body weight in multivariate analyses, and not with the measures of disease activity and functional limitations, in contrast to a recent study discussed below [13]. The overall results of this study therefore corroborate the view that there is not a strict relation between the measures of disease activity and functional limitations related to AS on one hand and cardiac manifestations on the other [9]. Whether cardiac conduction system abnormalities specifically or cardiac involvement in general is a spurious event in AS patients or determined by specific disease related or individual factors not included in this study remain to be elucidated. HLAB27 (mainly the subtype B270502) was present in a high proportion as expected, but the presence of one or two such alleles had no relation to the presence of cardiac conduction abnormalities.

The recent Dutch study reported associations between the PQ interval and age, disease duration, and BMI as well as between the QRS duration and male gender, disease duration and BASMI [13]. There were thus both similarities (e.g. similar proportion of patients with conduction abnormalities) and differences compared to the results of our study. Some differences might at least partly be due to selection. The Dutch study group consisted of two differently selected subgroups; one comprised AS patients with a diagnose duration of < 2 years and the other of AS patients with active disease starting up with tumour necrosis factor-α inhibitors. In contrast, our study group represents a larger and relatively unselected cohort of AS patients attending hospital based rheumatology clinics with a wider spectrum of disease duration. Our patients were also on average 10 years older and had a slightly higher proportion of women. The correlation between signs of disease activity and cardiac involvement in the Dutch study is therefore likely an effect of a highly selected and less representative cohort of AS patients than in the present study.

Is there a difference between AS and the other spondylarthropathies with regard to cardiac involvement, which can precede joint involvement in the setting of spondylarthropathies and even occurred without such involvement in the presence of HLA B27 in a man with two brothers who had typical AS [9,16]? From the wider perspective of all spondylarthropathies the occurrence of typical cardiac manifestations seems to be both intermittent (and transient) and often without relation to joint manifestations [9]. Even though our results do not exclude such a relation in AS patients, if it exists it is presumably weak. Whether analysis of modern inflammatory markers or components can distinguish between AS patients or HLA B27 positive individuals with/without a propensity for cardiac involvement remains to be elucidated. One methodological issue of concern is how to assess in quantitative terms the degree of inflammatory activity over time and put in relation to clinical phenomena that also might vary. This problem is obviously common to all rheumatic diseases and also e.g. to stress and its relation to cardiovascular risk.

HLA B27 subtypes have been studied in relation to other disease manifestations, but not previously reported for cardiac involvement [17]. HLA B2705 was the overwhelmingly dominant subtype in this cohort, and regarded as the genetic ancestor from which the other B27 alleles have evolved [17]. There was no statistically significant difference regarding B27 in patients with (20 of 21) vs. without (163 of 189) cardiac conduction abnormalities.

### Methodological aspects and limitations

This is a cross-sectional study focusing on ECG findings and any relation to disease markers and not a prevalence study, which would have required both a longitudinal design and a control group. We learnt two things > 30 years ago, when an AS cohort of 68 patients were studied by collection of all available ECG’s during 25 years: a high proportion (33%; 95% CI: 22–44) of patients had cardiac conduction abnormalities at any time applying conservative criteria, and even the most severe conduction abnormality 3^rd^ degree AV-block could be intermittent [18]. The present study has no age and sex matched population based control group. However, as “second best” alternative we compared with a recently acquired population sample of 547 subjects, men (n = 262) and women (n = 285) between 50 and 65 years (mean 58; SD 4 years), and found that 1^st^ degree AV block was > 4 times more common among our AS patients independent of which criteria were applied (p < 0.05, chi-square test).

One-hundred and fifty-one out of 361 AS patients declined to participate in the study. The most common reasons were declining participation or not responding to the invitation. These 151 patients were on average slightly younger, but the sex distribution was the same among included and not included patients. Since about 60% of eligible patients were included and the sex-distribution did not differ we do not believe that this limitation should substantially influence our findings.

Hypertension and coronary disease were found in similar proportions of AS patients with and without conduction abnormalities and none of our AS patients with conduction abnormalities had diabetes or hyperlipidemia. Therefore no adjustments for these variables have been applied in the statistical analyses. Because there is a known relation between aortic and mitral valvular disease and AS it was no surprise that this finding was over-represented among those with cardiac conduction abnormalities.

BASMI measures most of all spinal mobility and both disease and aging related factors might contribute to a higher score. BASFI and BASDAI on the other hand are based on the patients’ report on the ability to perform daily physical activities and their perception of symptoms, respectively [15]. In a cross-sectional study, when patients are not seen because they seek medical care due to worsening of the disease, these two latter scores naturally vary.

In this study we focused on patients fulfilling the modified New York criteria’s for AS but in the future it would also be of interest to assess whether or not cardiac conduction disturbances are evenly distributed across different phenotypes of spondylarthropathies.

### Implications

Does a prolonged PQ-interval matter? It is certainly not an indication for permanent pacemaker therapy. Recent studies have, however, pointed to an increased risk for atrial fibrillation (which might be intermittent or “paroxysmal” and easily overlooked) in patients with a prolonged PQ-interval or 1^st^ degree AV-block [19,20]. Mechanistically there seems to be a relation not only to impaired ventricular filling (preload) but also to the occurrence of mitral valve insufficiency with possible secondary effects on both atrial and ventricular structure and function [21]. Atrial fibrillation in turn is associated with a 5-fold increased risk for stroke, a doubled mortality risk and an increased risk for heart failure apart from reduced quality of life [22].

A complete LBBB (and other intraventricular conduction disturbances) may cause ventricular dyssynchrony with delayed left ventricular activation especially of the lateral basal parts and contribute to the development of heart failure, sometimes amenable to pharmacologic therapy and if needed cardiac resynchronisation therapy with right and left ventricular pacing [23]. Typical AS (or HLA B27) associated cardiac conduction system abnormalities are not only common but might thus even in seemingly less severe forms lead to clinically significant cardio-vascular morbidity, an issue which seems to have been forgotten in the new-born wake of interest for inflammation and cardiovascular risk [1-3].

Cardiac involvement is common in AS and other spondyloarthropathies and ECG is therefore suggested to be part of the routine evaluation of such patients, in particular when the patient presents with unspecific symptoms such as tiredness/fatigue, dyspnoea, and decreased physical capacity which might be related to cardiac conduction abnormalities and arrhythmias.

## Conclusions

Cardiac conduction abnormalities were common in this cross-sectional study but mainly associated with factors unrelated to rheumatic disease activity. Even relatively mild conduction system abnormalities might, however, have hemodynamic consequences of clinical importance.

## Abbreviations

ACEI: ACEI angiotensin converting enzyme inhibitor; ARB: Angiotensin II receptor blocker; AS: Ankylosing spondylitis; ASA: Acetylsalicylic acid; AV: Atrio-ventricular; BASDAI: Bath ankylosing spondylitis disease activity index; BASFI: Bath ankylosing spondylitis functional index; BASMI: Bath ankylosing spondylitis metrology index; BB: Beta-blocker; BMI: Body mass index; CAD: Coronary artery disease; CI: Confidence interval; CVD: Cardio-vascular disease; DMARD: Disease modifying antirheumatic drug; ECG: Electrocardiography; HLA: Human leukocyte antigen; HT: Hypertension; IVCD: Intra-ventricular conduction disturbances; LAFB: Left anterior fascicular block; LBBB: Left bundle branch block; NA: Not available; NSAID: Non-steroidal anti-inflammatory drugs; OR: Odds ratio; PM: Pacemaker; RBBB: Right bundle branch block; SD: Standard deviation.

## Competing interests

The authors declare that they have no competing interests.

## Authors’ contributions

HF-d’E conceived the study, participated in its design and interpretation of data and helped to draft the manuscript, HW participated in acquisition and interpretation of data and have been involved in drafting the manuscript, HC participated in study design, interpretation of data and revision of the manuscript, EK participated in study design, acquisition of data, interpretation of data and revision of the manuscript, LB participated in acquisition and interpretation of data and drafted the manuscript. All authors read and approved the final manuscript.

## Pre-publication history

The pre-publication history for this paper can be accessed here:

http://www.biomedcentral.com/1471-2474/14/237/prepub
